# Enhanced Production of IL-10 in PCR-Positive Dogs Infected with *E. canis* and *A. phagocytophilum* Facilitate Specific Immune Responses

**DOI:** 10.3390/microorganisms12122516

**Published:** 2024-12-06

**Authors:** Iskren Stanilov, Krasimira Gospodinova, Vladimir Petrov, Lyuba Miteva, Ilia Tsachev, Spaska Stanilova

**Affiliations:** 1 Department of Hygiene, Epidemiology, Microbiology, Parasitology and Infectious Diseases, Medical Faculty, Trakia University, 6000 Stara Zagora, Bulgaria; iskren.stanilov@trakia-uni.bg; 2 Department of General and Clinical Pathology, Faculty of Veterinary Medicine, Trakia University, 6000 Stara Zagora, Bulgaria; krasimira.gospodinova@trakia-uni.bg; 3 Department of Veterinary Microbiology, Infectious and Parasitic Diseases, Faculty of Veterinary Medicine, Trakia University, 6000 Stara Zagora, Bulgaria; vladimir.petrov@trakia-uni.bg (V.P.); iliya.tsachev@trakia-uni.bg (I.T.); 4 Department of Molecular Biology, Immunology and Medical Genetics, Medical Faculty, Trakia University, 6000 Stara Zagora, Bulgaria; lyuba.miteva@trakia-uni.bg

**Keywords:** cytokine, IL-10, Anaplasmataceae, antibody synthesis, Ehrlichia, Anaplasma, *E. canis*, *A. phagocytophilum*

## Abstract

Infection of dogs with the tick-borne rickettsiae *Ehrlichia* and *Anaplasma* provokes an immune response mediating the pathology and bacterial resistance. IL-10 is the main anti-inflammatory cytokine and plays a multifaceted role in host protection. The study aimed to investigate circulating IL-10 in 32 dogs naturally infected with *A. phagocytophilum* and *E. canis*, identified by PCR positivity, and 33 PCR-negative animals which tested positive for antibodies against these pathogens, as well as 22 healthy animals. The highest quantity of IL-10, measured by ELISA, was observed among dogs positive simultaneously for anti-*E. canis* and anti-A. *phagocytophilum* IgG antibodies, followed by dogs positive for anti-*E. canis* only. The concentration of IL-10 in PCR-positive dogs was almost three and a half times higher than that measured in the control group (77.09 ± 23.61 pg./mL vs. 21.55 ± 4.61 pg./mL; *p* = 0.0015) and five times higher than the concentration of interleukin in PCR-negative animals (77.09 ± 23.61 pg./mL vs. 14.86 ± 3.01 pg./mL; *p* = 0.000016). The highest level of IL-10 was observed in PCR-positive dogs with mixed infection (120.54 ± 44.18), followed by the level in PCR-positive dogs for *E. canis* only (78.81 ± 16.92). The lowest level of IL-10 was observed in PCR-positive dogs for *A. phagocytophilum* only (56.32 ± 12.68). We may suggest that infection with *E. canis* and *A. phagocytophilum* stimulates the IL-10 production in dogs, which may facilitate specific antibody responses.

## 1. Introduction

The most important tick-borne bacterial pathogens include mainly species belonging to the families of Gram-negative eubacteria such as Spirochaetaceae, Rickettsiaceae, and Anaplasmataceae. Rickettsiaceae and Anaplasmataceae are members of the order Rickettsiales [1]. While Spirochaetaceae (*Borrelia burgdorferi s.l*.) and Rickettsiaceae are the most common in humans, bacteria from the Anaplasmataceae family are essential in dogs with the tick-borne disease.

Bacteria of the Anaplasmataceae family belong to five genera: *Ehrlichia*, *Anaplasma*, *Neoehrlichia*, *Wolbachia*, and *Neorickettsia* [2]. The genera *Ehrlichia*, *Anaplasma*, and *Neoehrlichia* are transmitted by ticks, while the genera *Neorickettsia* and *Wolbachia* have different transmission mechanisms that do not involve ticks [2]. These bacteria are responsible for major endemic and emerging infectious diseases in animals and humans with significant economic and public health impacts [3].

Dogs acquire infections mainly with selected species from genera *Anaplasma* and *Ehrlichia,* including *A. phagocytophilum*, *E. canis*, *E. chaffeensis*, *E. ewingii*, and *A. platys* [4,5,6,7]. Over the past years, it has been reported that *E. canis* and *A. phagocytophilum* are the most emergent vector-borne pathogens in dogs in Bulgaria [5].

These intracellular pathogens parasitize in granulocytes (*A. phagocytophilum*) mononuclear leukocytes (*E. canis* and *E. chaffeensis*) and platelets (*A. platys*) [8,9,10,11].

Infection with rickettsiae *Ehrlichia* and *Anaplasma* provokes an immune response in the host, initiated by the binding of TLRs and mainly NLRs to the pathogen [6,12,13]. Several studies have confirmed the role of both innate and adaptive immune responses in host protection, mediating the pathology and bacterial resistance [14,15,16,17]. As the main regulators of the immune response, cytokines play a substantial role in modulating the protective host immune response. Previous results indicate that the role of IFN-γ, as the main cytokine of innate immunity mediating the Th1 cellular immune response, is involved in early control and clearance of these intracellular pathogens, although mice deficient in IFN-γ can survive [14,18]. 

The development of an inflammatory response against both *E. canis* and *A. phagocytophilum* is mediated by increased secretion of pro-inflammatory cytokines. These are also responsible for histopathological lesions in the host and the severity of the clinical manifestations [16]. Dysregulation of pro- and anti-inflammatory cytokine production following infection leads to the long-term persistence of these pathogens and potentiates chronic disease development. Given the essential requirement for anti-inflammatory cytokines, such as IL-10, we hypothesize that IL-10 secretion may be a clinically relevant feature. A key anti-inflammatory cytokine IL-10 is produced following experimental and clinical anaplasmosis and ehrlichiosis, and it has been linked to suppression of Th1 cell-mediated immunity during infection by inhibition of IFN-γ secretion via phagocytic cells [14,19]. IL-10 plays a multifaceted role in host protection and indirectly promotes humoral immunity by modulating B cell function; however, the role of IL-10 during infection with Anaplasmataceae family pathogens is not well elucidated.

The study aimed to investigate the concentration of circulating IL-10 in dogs naturally infected with pathogens from Anaplasmataceae, focusing on species, PCR results, and monospecific IgG results by ELISA.

## 2. Materials and Methods

### 2.1. Study Subjects and Blood Samples

This study included 87 privately owned dogs of both sexes, weighing 3 to 35 kg, divided into two groups: 65 patients of the “Small animal clinic” at the University Veterinary Hospital of Trakia University and 22 healthy animals with the absence of clinical signs of tick-borne and infectious diseases. The consent of the owners was obtained for all investigations carried out. The 65 animals exhibited characteristic symptoms of tick-borne disease, which was the reason for the inclusion of animals in the study. Criteria for inclusion were the presence of at least two characteristic clinical signs of monocytic ehrlichiosis or granulocytic anaplasmosis, such as fever, anorexia, depression, lethargy; enlarged lymph nodes and/or spleen, anemia, weight loss, hemorrhagic tendency, polyarthritis, CNS sigh, renal failure, thrombocytopenia, leukocytopenia, and/or pancytopenia. 

Blood samples from dogs were obtained from the cephalic vein using vacuum containers with EDTA as an anticoagulant. Part of the collected blood was centrifuged, and the separated plasma was used for serological studies, and another part was used to isolate DNA for PCR analysis. 

### 2.2. Ethical Approval

The study was approved by the Local Ethics Committee of the Faculty of Veterinary Medicine, Trakia University (FVM-09/ protocol 9 June 2020). Informed consent was obtained from the owners of all dogs involved in the study.

### 2.3. Rapid Diagnostic Test

The isolated dog plasma was tested with the rapid diagnostic kit SNAP4Dx Plus (IDEXX Laboratories, Westbrook, ME, USA). This kit is designed for the simultaneous detection of antibodies to *A. phagocytophilum*/*A. platys*, *E. canis*/*E. ewingii*, *B. burgdorferi*, and *Dirofilaria immitis* antigen for testing in veterinary clinics, characterized by a sensitivity of over 93% and specificity of over 98%) [20]. All samples were negative for *Borrelia burgdorferi* antibodies and *D. immitis* antigen, and the dogs were free of microfilariemia.

### 2.4. DNA Extraction

In the present study, the High Pure PCR Template Preparation Kit (Roche Diagnostics, Mannheim, Germany) was used for DNA isolation from 200 µL of canine venous blood following the manufacturer’s instructions. The extracted DNA was stored at −20 °C until PCR tests were performed. Spectrophotometric analysis by Biodrop Ulite+ (Biochrom, Cambridge, UK) was used to determine the purity and concentration of the extracted DNA samples.

### 2.5. PCR Detection of Pathogens

As the first step of our study, screening conventional PCR assay was performed on all DNA samples for amplification of the consensus 345 base pair (bp) sequence of the ribosomal gene *16S rRNA*, specific for bacterial species of the Anaplasmataceae family [21]. The primers and PCR conditions were presented previously by Stanilov et al., 2023 [22].

All positive samples from the above PCR assay were tested again with PCR using a species-specific primer set to amplify nucleotide sequences characteristic of *A. phagocytophilum* (444 bp amplicon from *AnkA* gene) or *E. canis* (409 bp amplicon from *16S rRNA*) [23,24]. The primers and PCR conditions were presented previously by Stanilov et al., 2023 [22].

### 2.6. ELISA Tests

Enzyme-linked immunosorbent assay (ELISA) was performed using ELISA kits, and the assay was performed according to the protocols provided by the manufacturer. The intensity of the color reaction was measured on a spectrophotometer ELISA reader (Rosys Anthos 2010, Wals, Austria) at a wavelength of 450 nm. The reaction was measured in units of optical density—OD units (optical density).

For the quantification of canine IL-10 in dog plasma, we used the Assay Genie Sandwich ELISA kit (Assay Genie, Dublin, Ireland) with high sensitivity and specificity for detection of Canine IL-10 in serum and plasma. The sensitivity of the test is <9.375 pg/mL and the range is from 15.625 to 1000 pg/mL. 

Determination of specific IgG antibodies in dog serum and plasma against *E. canis* or *A. phagocytophilum* was performed by ELISA kits EUROIMMUN (Medizinishe Labdiagnostika AG, Luebeck, Germany). After reading the optical density (OD), the calculation of the results is a ratio of the sample’s OD to the calibrator’s OD. The interpretation is as follows: a ratio < 0.8 is considered the absence of antibodies; a ratio of 0.8 to 1.1 is considered borderline, and a repeat sample is recommended after a week; and a ratio ≥ 1.1 is considered a positive sample for IgG antibodies against the respective pathogen.

### 2.7. Statistical Analysis

The statistical analysis of the data was performed with SPSS version 29.02 for Windows (SPSS Inc., Chicago, IL, USA), as well as with the indicated available online calculators StatPages.net website http://statpages.org/index.html, accessed on 2 June 2024, when appropriate.

The comparison of mean cytokine concentrations between groups of tested animals was performed by the Kruskal–Wallis test and subsequently by Mann–Whitney U tests. ANOVA variance analysis (post hoc multiple comparisons with LSD) was also used. Differences are considered statistically significant at *p* < 0.05.

## 3. Results

### 3.1. Concentration of IL-10 in Seropositive Samples, Tested for Specific Antibodies with Rapid Diagnostic Test and ELISA

The included 87 dogs were tested for specific antibodies against *E. canis* or *A. phagocytophilum* by the rapid SNAP4Dx Plus diagnostic test. The results showed 65 dogs positive for antibodies against *E. canis* and/or *A. phagocytophilum*, which confirm the exhibited clinical signs of monocytic ehrlichiosis or granulocytic anaplasmosis. The remaining 22 dogs were negative for antibodies against *E. canis* and/or *A. phagocytophilum* by the rapid SNAP4Dx Plus diagnostic test and were selected as the control group.

ELISA tests for detection of the anti-*E. canis* IgG antibodies and anti-*A. phagocytophilum* IgG antibodies in the obtained plasma samples were performed. Seven dogs positive for antibodies according to the rapid test are negative for IgG, which suggests early infection.

Table 1 presents the concentration of IL-10 measured by ELISA according to the positivity of the antibodies against *E. canis* and/or *A. phagocytophilum*. 

The post hoc test after ANOVA with multiple comparisons (LSD), performed with data from Table 1, revealed the following significance. The highest quantity of IL-10 was observed among cases positive simultaneously for anti-*E. canis* and anti-*A. phagocytophilum* antibodies (66.62 ± 25.11 vs. 21.55 ± 4.61 pg/mL; *p* = 0.22). IL-10 plasma levels were significantly higher in cases positive for anti-*E. canis* antibodies compared with control dogs (51.25 ± 10.94 vs. 21.55 ± 4.61 pg/mL; *p* = 0.41) in contrast to the cases positive for anti-*A. phagocytophilum* antibodies (28.34 ± 8.32 vs. 21.55 ± 4.61 pg/mL; *p* = 0.68). In addition, the differences in circulating IL-10 between groups of Anaplasmosis dogs and groups with mixed infection are with borderline significance (28.34 ± 8.32 vs. 66.62 ± 25.11; *p* = 0.053). 

Stratification according to the IgG antibodies revealed a similar tendency, with a significant increase in plasma IL-10 in the group with mixed infection against the group of animals infected with *A. phagocytophilum* only (67.14 ± 19.87 vs. 15.35 ± 3.73 pg/mL; *p* = 0.009), as well as against the control group (67.14 ± 19.87 vs. 21.55 ± 4.61 pg/mL; *p* = 0.01). The differences in circulating IL-10 between the group of dogs positive for anti-*A. phagocytophilum* IgG and the group with anti-*E. canis* IgG antibodies are with borderline significance (15.35 ± 3.73 vs. 49.52 ± 11.55 pg/mL; *p* = 0.055). 

### 3.2. Concentration of IL-10 According to Results from PCR Tests for the Pathogens of Anaplasmataceae Family

DNA samples were isolated from all 65 dogs, positive for antibodies against *E. canis* and/or *A. phagocytophilum* by the rapid SNAP test and from 22 control dogs. After performing the PCR test with the primers specific for the Anaplasmataceae family, we found thirty-two positive and thirty-three negative dogs among the dogs, showing a positive result of the antibody tests. The control animals are a group of healthy dogs, and they are negative by both SNAP and PCR tests. PCR (−) animals are positive by SNAP test. 

**Figure 1:**
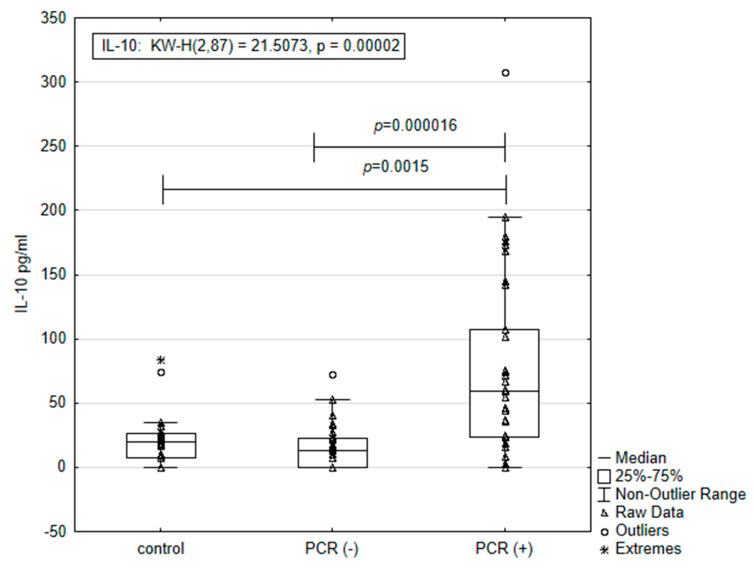
The quantity of IL-10 in dog plasma depends on the presence of tick-borne infection, detected by PCR assay with primers specific for the Anaplasmataceae family. PCR (+) animals are positive by both PCR and SNAP tests. PCR (−) animals are positive by SNAP test only. The control group includes healthy animals, negative by both tests used.

Independent Kruskal–Wallis test analysis of the results obtained from the IL-10 assay for the control (healthy animals), PCR-positive (n = 32), and PCR-negative animal (n = 33) groups showed statistically significant differences between groups (*p* = 0.00002). The results for the concentration of IL-10 in the PCR-positive animals, PCR-negative and SNAP 4DX-positive animals, and the control group are presented in Figure 1. 

The concentration (mean ± SE) of IL-10 in the group of PCR-positive dogs was almost three and a half times higher than that measured in the control group (77.09 ± 23.61 pg/mL vs. 21.55 ± 4.61 pg/mL; *p* = 0.0015; Mann–Whitney U test) and five times higher than the concentration of interleukin in the group of PCR-negative animals (77.09 ± 23.61 pg/mL vs. 14.86 ± 3.01 pg/mL; *p* = 0.000016; Mann–Whitney U test).

### 3.3. Concentration of IL-10 According to Results from Species-Specific PCR Tests for E. canis and A. phagocytophylum 

PCR positivity for Anaplasmataceae family dogs were further tested by PCR with species-specific primers for *E. canis* and *A. phagocytophylum*. When the groups of PCR-tested animals were separated, depending on the pathogen, statistically significant differences in IL-10 concentration were found between PCR-positive and PCR-negative animals for both groups, as well as compared to the group of healthy animals by the Kruskal–Wallis test. 

**Figure 2:**
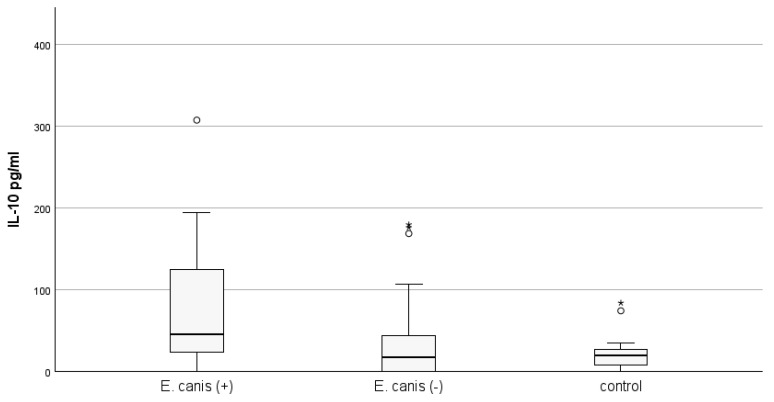
The quantity of IL-10 in dog plasma from the three groups depends on the presence of *E. canis* infection detected by PCR assay (*p* = 0.001; KW). *E. canis* (+) animals are positive by both *PCR* and SNAP tests. *E. canis* (−) animals are negative by PCR test, but positive by SNAP test. The control group included healthy animals negative by both tests used. Outliers (°) and extremes (*) are presented.

The concentration of IL-10 (mean ± SE) in the PCR-positive group for *E. canis* (n = 20) was statistically significantly higher than that measured in the control group (93.41 ± 18.81 vs. 21.55 ± 4.61 pg/mL; *p* = 0.001; Mann–Whitney U test), as well as in the PCR-negative group for *E. canis* animals (n = 45), but was positive according to the immunological test (93.41 ± 18.81 vs. 25.91 ± 4.82 pg/mL; *p* = 0.002). Differences in IL-10 concentration between *E. canis* PCR-negative and healthy animal groups did not reach statistical significance. The results are presented in Figure 2.

**Figure 3:**
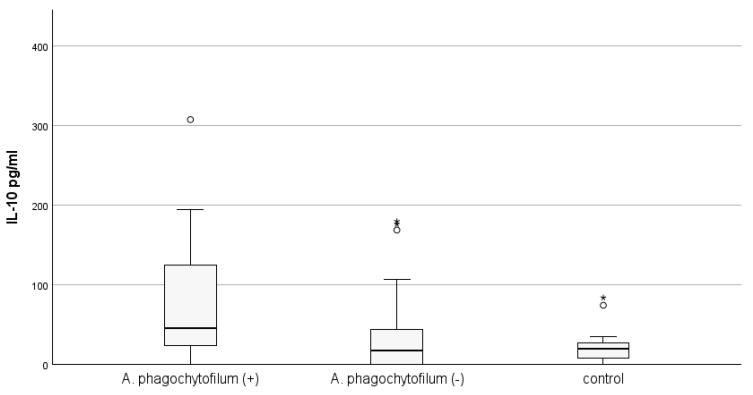
The quantity of IL-10 in dog plasma from the three groups depends on the presence of *A. phagocytophilum* detected by PCR assay (*p* = 0.013; KW). *A. phagocytophilum* (+) animals are positive by both PCR and SNAP tests. *A. phagocytophilum* (−) animals are negative by PCR test, but positive by SNAP test. The control group included healthy animals, negative by both tests used. Outliers (°) and extremes (*) are presented.

The mean concentration of IL-10 in the PCR-positive group for *A. phagocytophilum* (n = 19) was statistically significantly higher than that measured in the control group (79.98 ± 18.84 vs. 21.55 ± 4.61 pg/mL; *p* = 0.006; Mann–Whitney U test), as well as in the PCR-negative group for *A. phagocytophilum* animals (n = 46), but positive according to the immunological test (79.98 ± 18.84 vs. 32.93 ± 6.68 pg/mL; *p* = 0.009). Differences in IL-10 concentration between *E. canis* PCR-negative and healthy animal groups did not reach statistical significance. The results are presented in Figure 3.

The IL-10 quantity in the group of *E. canis*-positive dogs was greater compared to those positive for *A. phagocytophilum* without statistical significance (93.41 ± 18.81 vs. 79.98 ± 18.84; *p* > 0.05).

**Figure 4:**
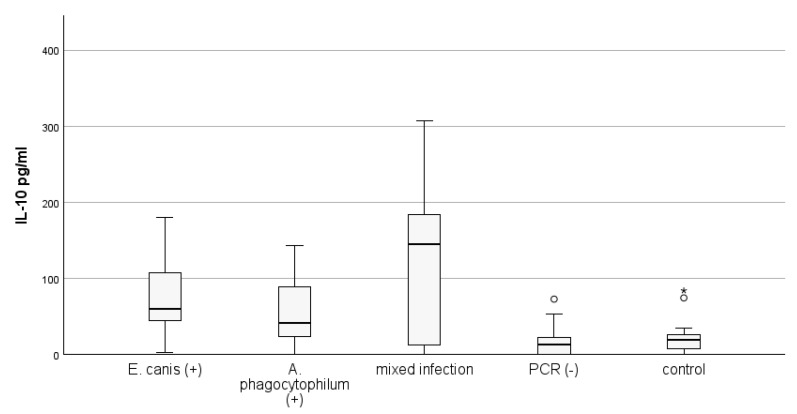
The quantity of IL-10 in dog plasma depends on the presence of *E. canis* alone; *A. phagocytophilum* alone and mixed infection detected by PCR assay (*p* < 0.001; KW). PCR (−) animals are positive by SNAP test only. The control group included healthy animals, negative by both tests used. Outliers (°) and extremes (*) are presented.

We also assessed the differences between circulating levels of IL-10 depending on the presence of single or mixed infection with two investigated pathogens, detected by PCR. Out of 32 PCR-positive dogs, thirteen were positive for *E. canis* only. Twelve dogs were positive for *A. phagocytophilum* only, and seven dogs had mixed infections, being simultaneously positive for both causative pathogenic agents. The results are presented in Figure 4.

The highest level of circulating IL-10 was observed in dogs infected with both pathogens (120.54 ± 44.18), followed by the level in dogs infected with *E. canis* only (78.81 ± 16.92). The lowest level of circulating IL-10 was observed in dogs infected with *A. phagocytophilum* only (56.32 ± 12.68). Statistical analysis shows a lack of significant increase in IL-10 between groups.

## 4. Discussion

The obtained results showed a significant increase in IL-10 in dogs infected with pathogens of the Anaplasmataceae family, confirmed by PCR test, not only compared to healthy animals but also compared to dogs positive only for pathogen-specific antibodies by SNAP test. A similar increase was observed when the animals were separated by PCR with species-specific primers for *E. canis* and *A. phagocytophilum*. A statistically significant higher IL-10 concentration was found for PCR-positive animals for any of the studied pathogens compared to the healthy animals and SNAP-positive dogs. Moreover, coinfected animals displayed the highest concentration of IL-10. A similar tendency was revealed when dogs were stratified by immunological tests for presence of pathogen-specific IgG. Collectively, these results allow us to hypothesize that the persistence of bacteremia may be the cause of increased IL-10 production in the host’s immune cells.

IL-10 plays an essential role in the regulation of macrophage and monocyte functions, participates in the balancing of Th1/Th2 mediated immune response, as well as in reducing the production of pro-inflammatory cytokines such as IL-1β, IL-6, IL-12, and TNF- α, including in dogs infected with bacteria from the Anaplasmataceae family [25]. In support of this hypothesis are the results of the experiments of Ismail and collaborators. They found elevated serum levels of IL-10 in mice after experimental infection with a virulent strain of *E. muris* at day 8 post-infection, and the production of high levels of the cytokine was mainly due to IL-10-producing lymphocytes and was antigen-specific [19]. Another study found that in dogs with experimental *Ehrlichia sp*. infection, there are characteristic dynamics in serum levels of TNF-α and IL-10 during the development of the disease [26]. The authors found that up to 18 days post-infection, there was a significant production of TNF-α and IL-10 levels from dogs’ leucocytes and splenocytes, but this difference disappeared after 30 days.

As is well known, the cytokines are the key regulators of type and protective features of host immune response developed after infection. Many studies have suggested that innate and adaptive cell-mediated immune responses play a pivotal role in the elimination of intracellular pathogens such as Anaplasmataceae species [27,28,29,30]. Although the cellular immune response is pivotal against intracellular pathogens, the humoral immune response is also activated, and the production of antibodies is essential to cope with infection. Experiments with mice protected by passive immunization became refractory to *A. phagocytophilum* infection, suggesting that antibodies are required against *A. phagocytophilum* [31,32]. In a recent study, experimentally infected dogs with Anaplasmataceae species—*E. canis*, *E. chaffeensis*, *A. platys*, and *A. phagocytophilum*—revealed a significant overlap in clinical, hematological, and pathological changes resulting from the infections, although they infect different type of blood cells. Anaplasmataceae pathogens induced IgG responses starting on day 7 post-infection, which was predominantly the Th1 type IgG2 [6]. Despite the development of an effective immune response in dogs, characterized by high titers of specific IgG2 antibodies and Th1-biased immune cells, complete pathogen elimination was not observed. The prolonged persistence of Ehrlichia and Anaplasma species in infected dogs is well documented [33,34,35]. Moreover, the histopathological damages and hematological modification in Anaplasmosis are related to exacerbated immune responses, mainly by inflammatory responses [36]. Canine ehrlichiosis can lead to a systemic inflammatory response syndrome, and assessing the intensity of inflammation is important to disease treatment [37]. Pro-inflammatory cytokines are strongly associated with the clinical aggravation of disease due to activation of inflammatory cells and induction of phagocytosis. *Anaplasma phagocytophilum* infection leads to the upregulation of pro-inflammatory cytokines TNF-α, IL-1, MIP-2, and IL-6 [38]. A similar pro-inflammatory cytokine profile is observed in the early stage of Ehrlichia infection [26]. 

Effective immunity against both Ehrlichia and Anaplasma species is mediated by CD4+ T lymphocyte-driven immune response and through the production of gamma interferon (IFN-γ) [39]. IFN-γ activates macrophage production of reactive oxygen species and nitric oxide, causing cytotoxic effects on intracellular pathogens and bacterial clearance. However, overproduction of IFN-γ mediated pathological changes in infected tissues [40]. In IFN-γ knockout mice, bacterial tissue levels are increased in the early phase of infection, but tissue damage is absent, and the bacteria are eventually eliminated [16]. The same study described enlarged lesions in IL10 knockout mice that showed normal levels of IFN-γ.

IL-10, as a main anti-inflammatory cytokine, ensures host protection from over-exuberant inflammatory responses to pathogens. The anti-inflammatory activity of interleukin 10 is mediated by different mechanisms, including suppression of Th1cytokine synthesis [41]. In addition, IL-10 is known as a cytokine with a double-edged role in the immune response to intracellular pathogens [42,43]. In early infection, IL-10 may inhibit the production of pro-inflammatory cytokines, boost bacterial dissemination to the bloodstream, and could be exploited by pathogens to facilitate their survival. During the development of the immune response, this cytokine can significantly enhance the host’s specific immune response to bacteria. Significantly higher IL-10 levels in dogs with current infection with intracellular pathogens from the Anaplasmataceae family, confirmed by PCR-positive results compared with PCR-negative dogs, suggested that the production of the IL-10 in infected dogs can be stimulated by infected cells, monocytes, and neutrophils by the pathogens, but can also be as a defense immune response of the host. Also, we found the highest quantity of IL-10 in dogs positive simultaneously for anti-*E. canis* and anti-*A. phagocytophilum* IgG antibodies. Taken together with the above data, a protective role for IL-10 in limiting infection of Anaplasmataceae pathogens could be proposed. 

However, according to the study by Lima et al., there was no significant difference in serum levels of TNF-α and IL-10 between limited groups of naturally infected *Ehrlichia* sp. dogs, patients of the veterinary clinic, and the control group of uninfected animals [44]. This result could be explained by the time of infection, bearing in mind that the time of naturally infected dogs cannot be precisely determined, which may have influenced the results. Experimentally, the course of *E. canis* infection can be sequentially divided into acute, subclinical, and chronic phases, and the differences in immune markers between the phases are observed. do Carmo et al. reported that IL-10 levels were lower in dogs who experienced the acute phase than in dogs in the subclinical form of canine ehrlichiosis [45]. 

Several studies have reported variable hematological alternations in cases with ehrlichiosis and anaplasmosis, from leukopenia to leukocytosis, neutropenia, neutrophilia, lymphopenia/lymphocytosis, monocytosis, eosinophilia, and thrombocytopenia [46]. Because of this, these hematological parameters are not sufficient to confirm the diagnosis [47]. PCR is more sensitive than microscopic examination of morulae for detection of current infection, and serology is useful for a presumptive diagnosis. 

In addition to being anti-inflammatory, IL-10 also has an immunoregulatory role, particularly by stimulating the proliferation of activated B lymphocytes. Several studies have reported that acute Ehrlichiosis and Anaplasmosis are associated with a reduced number and activities of B lymphocytes to differentiate and produce pathogen-specific antibodies [18,38,48]. The observed elevated IL-10 in dogs, which were positive for anti-*E. canis* and anti-*A. phagocytophilum* IgG antibodies in mono-infection and co-infection, showed the potential role of this cytokine in the host’s immune response to foster recovery from infection. A similar observation has been made by Guthmiller et al. The authors found that IL-10 is essential for the generation of germinal center B cell responses, and humoral immunity against plasmodium [49]. In support of the protective role of IL-10 after infection with Anaplasmataceae are the results of a recent study, which presented data on an increase in the concentration of serum IL-10 in dogs with ehrlichiosis after treatment with doxycycline [50].

A limitation of this study is the absence of data on the onset of infection, as well as the performance of more than one measurement of IL-10 to follow the dynamics of the production of this cytokine. Further studies are needed to clarify the mechanism of IL-10 involvement in IgG synthesis during the infection with *E. canis* and *A. phagocytophilum*. 

## 5. Conclusions

In summary, our data showed significant elevation in plasma IL-10 in dogs infected with pathogens of the Anaplasmataceae family, confirmed by PCR test, particularly *E. canis* and *A. phagocytophilum*. Moreover, coinfected animals displayed the highest concentration of IL-10. Also, we found the highest quantity of IL-10 in dogs positive simultaneously for anti-*E. canis* and anti-*A. phagocytophilum* IgG antibodies. The observed elevated IL-10 in dogs, which were positive for anti-*E. canis* and anti-*A. phagocytophilum* IgG antibodies in mono-infection and co-infection, showed the potential role of cytokine in the host’s immune response to foster recovery from infection. Taken together with the above data, a protective role for IL-10 in limiting infection of Anaplasmataceae pathogens could be proposed. We may suggest that infection with *E. canis* and *A. phagocytophilum* stimulates the IL-10 production in dogs, which may facilitate specific antibody responses.

## Figures and Tables

**Table 1:** Concentration of IL-10 in dogs according to the positivity of the antibodies test.

Cases (n = 65)	SNAP Test		IgG Antibodies by ELISA	
	n (%)	IL-10 pg/mL mean ± SE	n (%)	IL-10 pg/mL mean ± SE
Positive for *E. canis* only	32 (49.2)	51.25 ± 10.94	27 (41.5)	49.52 ± 11.55
Positive for *A. phagocytophilum* only	21 (32.3)	28.34 ± 8.32	14 (21.5)	15.35 ± 3.73
Positive for *E. canis* and *A. phagocytophilum*	12 (18.5)	66.62 ± 25.11	17 (26.2)	67.14 ± 19.87
All positive for *E. canis*	44 (67.7)	55.44 ± 10.38	44 (67.7)	56.33 ± 10.39
All positive for *A. phagocytophilum*	33 (50.8)	42.26 ± 10.81	31 (47.7)	43.75 ± 11.85
Cases with clinical symptoms negative for both pathogens	0	-	7 (10.8)	48.70 ± 21.14

The concentration of IL-10 (mean ± SE) measured in the control (healthy) group of dogs (n = 22) is 21.55 ± 4.61 pg/mL.

## Data Availability

The data presented in this study are available upon request from the corresponding author due to privacy.
